# Long-term follow-up of patients with drug-related atrioventricular block without a need of permanent pacemaker during index hospitalization

**DOI:** 10.1186/s43044-022-00297-3

**Published:** 2022-08-01

**Authors:** Kemal Emrecan Parsova, Mert Ilker Hayiroglu, Levent Pay, Goksel Cinier, Kadir Gurkan

**Affiliations:** 1 Department of Cardiology, Zile State Hospital, Tokat, Turkey; 2 Department of Cardiology, University of Health Sciences, Dr. Siyami Ersek Thoracic and Cardiovascular Surgery Training and Research Hospital, Selimiye Mahallesi, Tıbbiye Caddesi, No: 13, Uskudar, 34668 Istanbul, Turkey

**Keywords:** Atrioventricular block, Permanent pacemaker, Glomerular filtration rate

## Abstract

**Background:**

Most of the patients hospitalized due to drug-related atrioventricular (AV) block do not require permanent pacemaker implantation (PPI) since AV block regresses following cessation of the responsible drug. However, AV block requiring PPI may relapse in long-term follow-up. In this study, we retrospectively evaluated the factors predicting the need for a PPI in the long-term follow-up in patients admitted to our hospital with drug-related AV block but did not require PPI in index hospitalization.

**Results:**

We evaluated 177 patients who had been hospitalized with drug-related AV block between January 2012 and July 2020 and who had not required PPI during hospital follow-up. The patients were divided into two groups according to whether PPI was performed or not. The independent predictors of long-term PPI were evaluated and the effect of glomerular filtration rate (GFR) of the patients during the index hospitalization on the long-term outcome was compared. A GFR above 60 ml/min is an independent significant risk factor in predicting long-term permanent pacemaker implantation in drug-related AV blocks. It is found that the need for PPI was 2.64 times higher without adjusted and 1.9 times higher with adjusted for all covariates in patients with GFR above 60 ml/min during hospitalization compared to those with GFR below 30 ml/min.

**Conclusions:**

GFR may be considered as an indicator of the PPI need in patients with drug-related atrioventricular AV block.

## Background

There are several congenital or acquired etiological factors that can deteriorate the atrioventricular (AV) conduction system and lead to AV block. Acquired causes are more common than congenital causes. Acquired causes include degenerative, iatrogenic, infectious, inflammatory or ischemic processes. In some acquired etiologies such as drug use, thyroid dysfunction, electrolyte disorders, patients' AV blocks may regress as a result of discontinuation of the drug responsible for the block or treatment of hypothyroidism, hyperthyroidism or electrolyte disorder and pacemaker (PM) needs is obviated during index hospitalization. Despite the discontinuation of the responsible drug in some patients, AV block reoccurs in the long-term follow-up of the patients, and these patients inevitably need permanent pacemaker implantation (PPI) with postponement from the index hospitalization. Studies have shown that regression in AV block is 41–71% after discontinuation of the responsible drug in drug-induced AV blocks [1].

Potassium is vital for regulating the normal electrical activity of the heart. Hyperkalemia affects the AV conduction system and leads to blockage in the AV conduction system, manifested by prolongation of the His-Ventricle interval and widening of the QRS wave. Potassium excretion is impaired in patients with chronic kidney failure and blood potassium level may increase. Chronic kidney failure leads to an increase in the plasma half-life of a number of beta blocker molecules; thus, even at therapeutic doses, the plasma beta blocker molecule level can be higher than normal. Therefore, there may be a tendency for drug-related AV blocks in patients with chronic renal failure, with both electrolyte disturbances and changes in drug metabolism. However, we do not have enough data about the long-term effect of glomerular filtration rate (GFR) on drug-related AV blocks since the effect of GFR on the conduction system has not been elucidated in patients with drug-related AV block [2–4].

In this study, we retrospectively evaluated the factors predicting the need for PPI in the long-term follow-up of patients who were admitted to our hospital with drug-related AV block but did not need PPI.

## Methods

The study has a single center, cross-sectional and retrospective design. The approval for the study was obtained from the Ethics Committee (HNEAH-KAEK 2020/KK/223). The study population was enrolled by screening patients who were hospitalized with the diagnosis of AV block to our tertiary center between January 2012 and July 2020 and discharged without PPI following restoration of the sinus rhythm in the index hospitalization. If there was a right bundle branch block, left bundle branch block, left anterior hemiblock, left posterior hemiblock or combination of these rhythm disturbances in the restored sinus rhythm, electrophysiological study (EPS) was performed, and permanent pacemaker was implanted according to HV interval. The patients, who were evaluated with EPS, were excluded due to standardization of the study population. Demographic characteristics of all patients, comorbid diseases, baseline features and follow-up information after discharge were obtained using the hospital automation system and Ministry of Health system. Patients aged 18 and over who were hospitalized with second degree or higher AV block, were using a class I or class II or class III or class IV antiarrhythmic drug or digoxin regardless of the duration of use of the responsible drug, did not need PPI during hospital follow-up were included in this study. Permanent atrial fibrillation patients with a heart rate of less than 60 beats per minute and an equal distance between the QRS waves on the electrocardiogram (ECG) were considered AV block. Patients also were included according to availability of health system records on hospital automation system and the Ministry of Health system. Patients with acute myocardial infarction, AV block due to vasovagal reaction, sick sinus syndrome, periprocedural AV block (due to cardiac surgery, cardiac percutaneous interventions or radiofrequency ablation), digoxin toxicity (defined according to serum digoxin level or clinical complaints coherent with digoxin toxicity), and advanced AV block due to non-drug causes excluded from the study.

The patients' complete blood count, creatinine, glomerular filtration rate, urea, AST, ALT, sodium, potassium, calcium, fasting blood glucose, TSH, T3, T4, troponin I values are taken as the values in the first blood samples taken at the time of admission. Troponin I peak value is accepted as the highest troponin level in the blood samples taken at the hospitalization of the patient.

Digoxin toxicity is defined as the blood digoxin level above 2 nmol/l and the patient having complaints such as nausea, vomiting, color vision disorders or the blood digoxin level above 2.5 nmol/l without symptoms.

As echocardiography values that were obtained for the first time in the emergency department or coronary intensive care unit when the patient was admitted to the hospital were accepted.

The responsible drug was discontinued in all patients hospitalized with drug-related AV block. PPI was performed in patients whose block continued after the drug was discontinued and the sufficient half-life period passed or the block regressed during the hospitalization period and then the block was recurred. In the follow-up, patients whose AV conduction system recovered after the responsible drug was discontinued and whose block did not recur during hospitalization were followed up, and patients who did not need PPI were discharged. All patients included in the study, who did not need PPI during hospitalization, have a follow-up period of at least 6 months after discharge. PPI was applied to patients when the block recurred or persisted in the follow-up of patients whose drug was discontinued.

### Statistical analysis

The data analysis was performed using IBM SPSS Statistics for Windows, Version 20.0 (IBM Corp., Armonk, NY, USA) software. The Kolmogorov Smirnov test was used to test the distribution pattern. Data were presented as mean ± SD for normally distributed data, and as median (interquartile range) for continuous variables that were not normally distributed. The number of cases and percentages were used for categorical data. The mean differences between groups were compared using the Student’s *t* test. The Mann–Whitney *U* test was applied for comparisons of the data that were not normally distributed. Categorical data were analyzed with Fisher’s exact test when 1 or more cells had an expected frequency of 5 or less. Otherwise, Pearson’s chi-square test was applied. Variables that could be a predictor of long-term PPI and with a significant *p* value in Tables 1 and 2 were entered into univariate cox-regression analysis. Variables with a *p* value < 0.1 in univariate analysis were included into multiple cox-regression analysis. Three Cox multivariable models were also used in regard to glomerular filtration rate: model I, unadjusted; model II, medications adjusted; and model III, adjusted for age, gender, hypertension, diabetes mellitus, smoking, hyperlipidemia, chronic obstructive pulmonary disease, coronary artery disease, atrial fibrillation, chronic renal failure, medications, first measurement during hospitalization of the following laboratory values (creatinine, urea, peak troponin, potassium) and left ventricle ejection fraction. A *p* value < 0.05 was considered statistically significant.

**Table 1:** Comparison of demographic and clinical characteristics of patients according to permanent pacemaker implantation in the follow-up

	Permanent pacemaker implantation (−) in follow-up *n* = 128	Permanent pacemaker implantation (+) in follow-up *n* = 49	*p* value
Age (years)	76 (66–83)	73 (65–80)	0.286
Male gender	54 (42.2%)	21 (42.9%)	0.936
Hypertension	99 (77.3%)	42 (85.7%)	0.203
Diabetes Mellitus	62 (48.4%)	21 (42.9%)	0.505
Hyperlipidemia	29 (22.7%)	12 (24.5%)	0.797
Smoking	12 (9.4%)	2 (4.1%)	0.355
Chronic renal failure	45 (35.2%)	12 (24.5%)	0.167
Congestive heart failure	18 (14.1%)	8 (16.3%)	0.706
Chronic obstructive pulmonary disease	18 (14.1%)	3 (6.1%)	0.121
Alzheimer	6 (4.7%)	1 (2.0%)	0.675
Coronary artery disease	47 (36.7%)	20 (40.8%)	0.616
Percutaneous coronary intervention	28 (21.9%)	7 (14.3%)	0.245
Coronary artery bypass grafting	23 (18.0%)	8 (16.3%)	0.796
Myocardial infarction	37 (28.9%)	14 (28.6%)	0.965
Cerebrovascular accident	10 (7.8%)	1 (2.0%)	0.294
Paroxysmal atrial fibrillation	0 (0.0%)	1 (2.0%)	0.277
Permanent atrial fibrillation	26 (20.3%)	5 (10.2%)	0.098
Hospitalization period, days	7 (5–10)	9 (6–12)	0.186
Follow-up	33 (17–61)	52 (26–89)	
Time to permanent pacemaker implantation, months		10 (3–23)	

Continuous variables are presented as median (interquartile range)

Nominal variables presented as frequency (%)

**Table 2:** Classification of cases according to their ECG characteristics

	Permanent PM implantation in the follow-up (−) *n* = 128	Permanent PM implantation in the follow-up (+) *n* = 49	*p* value
Second-degree AV block	3 (2.3%)	1 (2.0%)	1.000
Mobitz Type 1 AV block	2 (1.6%)	1 (2.0%)	0.624
Mobitz Type 2 AV block	3 (2.3%)	1 (2.0%)	1.000
2:1 AV block	27 (21.1%)	15 (30.6%)	0.190
Third degree AV block	72 (56.3%)	31 (63.3%)	0.395
Atrial fibrillation and bradyarrhythmia	26 (20.3%)	6 (12.2%)	0.198

## Results

We evaluated retrospectively the patients who had been hospitalized with drug-related second degree or higher AV block between January 2012 and July 2020 at Dr. Siyami Ersek Thoracic and Cardiovascular Surgery Research and Training Hospital and who had not required permanent PM during hospital follow-up. 177 cases were included in the study. In 128 of the cases, there was no need for PPI in the follow-up after discharge, 49 of the cases (27% of the cases) required PPI in the follow-up after discharge. The demographic and clinical characteristics of the patients are summarized in Table 1.

Third degree AV block was the most frequently observed ECGs in the hospitalization (56.3% for those who did not require PPI in follow-up, 63.3% for those who required PPI in follow-up). The classification of the hospitalization ECGs of the cases is shown in Table 2.

Beta blockers are the most used drugs at home as risk factor drugs. (80.5% in those who did not require PPI in follow-up, 91.8% in those who required PPI in follow-up) 26.6% of the group that did not require PPI in follow-up and 10.2% of the group that required PPI in follow-up used 2 or more drugs deteriorating AV node. The characteristics of the cases regarding drug use are summarized in Table 3.

**Table 3:** Drug use characteristics of the cases

	Permanent PM implantation in the follow-up (−) *n* = 128	Permanent PM implantation in the follow-up (+) *n* = 49	*p* value
1 drug risk factor	94 (73.4%)	44 (89.8%)	0.013
> 1 drug risk factor	34 (26.6%)	5 (10.2%)	0.013
Calcium channel blocker	32 (25.0%)	5 (10.2%)	0.022
Digoxin	8 (6.3%)	1 (2%)	0.448
Beta blocker	103 (80.5%)	45 (91.8%)	0.053
Amiodarone	10 (7.8%)	0 (0.0%)	0.064

The laboratory and transthoracic echocardiography values of the cases at hospitalization are summarized in Table 4.

**Table 4:** Laboratory and transthoracic echocardiography values of the patients at hospitalization

	Permanent PM implantation in the follow-up (−) *n* = 128	Permanent PM implantation in the follow-up (+) *n* = 49	*p* value
Hemoglobin (g/dl)	11.6 (10.0–13.3)	11.9 (11.0–13.5)	0.120
White blood cells (cell/µl)	9.7 (7.7–12.0)	8.4 (7.3–10.7)	0.120
Lymphocyte (cell/µl)	1.7 (1.2–2.4)	2.0 (1.3–2.5)	0.146
Creatinine (cell/dl)	1.3 (0.9–1.9)	0.9 (0.7–1.4)	0.001
Urea (mg/dl)	28.0 (19.0–40.0)	23.0 (17.0–31.0)	0.059
Glomerular filtration rate (ml/min)	44.0 (28.0–75.0)	69.0 (41.0–89.0)	0.002
Glomerular filtration rate < 30 ml/min	38 (29.7%)	6 (12.2%)	0.012
Glomerular filtration rate 30–60 ml/min	46 (35.9%)	12 (24.5%)	0.140
Glomerular filtration rate > 60 ml/min	44 (34.4%)	31 (63.3%)	0.001
TSH (mIU/l)	1.3 (0.8–2.2)	1.4 (0.8–2.0)	0.496
T3 (pg/ml)	2.2 (1.8–2.6)	2.4 (2.0–2.7)	0.142
T4 (ng/dl)	1.0 (0.9–1.2)	1.0 (0.9–1.2)	0.851
Troponin I first admission (ng/ml)	0.01 (0.01–0.04)	0.02 (0.01–0.03)	0.584
Troponin I peak value (ng/ml)	0.04 (0.01–0.21)	0.03 (0.01–0.09)	0.072
Glucose (mg/dl)	129 (103–199)	123 (105–207)	0.880
ALT (IU/l)	24 (15–45)	24 (14–51)	0.846
AST (IU/l)	27 (19–55)	25 (17–39)	0.222
Sodium (mEq/l)	139 (135–141)	140 (136–141)	0.260
Calcium (mg/dl)	9.0 (8.5–9.5)	9.1 (8.6–9.5)	0.445
Potassium (mEq/l)	4.9 (4.3–5.6)	4.4 (4.2–5.1)	0.037
Left ventricle ejection fraction, %	60 (50–60)	60 (50–60)	0.296
LVEDD, mm	48 (45–52)	45 (42–54)	0.349
LVESD, mm	30 (26–35)	29 (26–34)	0.885
Left atrium anteroposterior diameter, mm	41 (34–45)	38 (36–42)	0.804

Continuous variables are presented as median (interquartile range). Nominal variables presented as frequency (%)

*LVEDD* left ventricular end-diastolic diameter, *LVESD* left ventricular end-systolic diameter

**Fig. 1:**
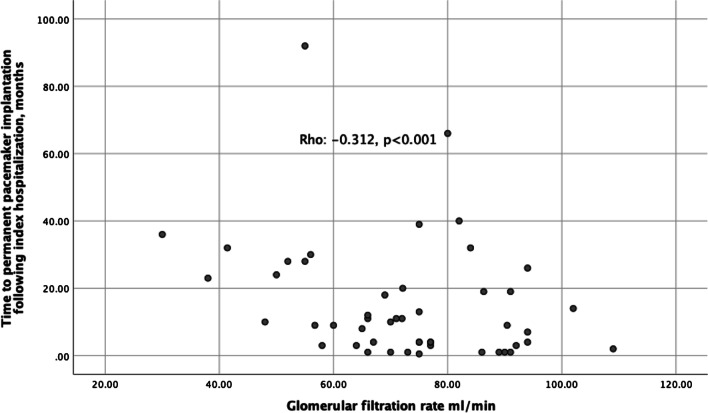
Correlation analysis. The need for PPI was higher in patients with GFR above 60 ml/min during hospitalization compared to those with GFR below 30 ml/min

A GFR above 60 ml/min is an independent significant risk factor in predicting long-term permanent pacemaker implantation in drug-related AV blocks based on univariate analysis (*p* = 0.001). A GFR above 60 ml/min is a significant risk factor, independent of the use of more than 1 drug and calcium channel blocker in index hospitalization, according to multivariate analysis (*p* = 0.021). It is found that the need for PPI was 2.64 times higher without adjusted and 1.9 times higher with adjusted for all covariates in patients with GFR above 60 ml/min during hospitalization compared to those with GFR below 30 ml/min (Fig. 1, Tables 5, 6).

**Table 5:** Univariable analysis and multivariable model for long-term permanent pacemaker implantation prediction according to admission demographic and clinical characteristics, laboratory parameters and echocardiography variables

Univariable analysis	*p* value	HR (95% CI)	Multivariable analysis	*p* value	HR (95% CI)
Beta blocker	0.056	2.714 (0.973–7.569)			
BUN	0.059	0.981 (0.962–1.001)			
Troponin peak	0.078	0.071 (0.004–1.346)			
> 1 drug usage	0.009	0.210 (0.065–0.674)	> 1 drug usage	0.009	0.358 (0.167–0.771)
Calcium channel blockers	0.023	0.342 (0.135–0.862)			
Creatinine	0.026	0.598 (0.380–0.939)			
Potassium	0.036	0.711 (0.517–0.978)			
GFR > 60	0.001	2.680 (1.497–4.798)	GFR > 60	0.003	2.472 (1.357–4.500)

All clinically relevant parameters were included in the model. HR, Hazard ratio; CI, confidence interval

**Table 6:** Cox proportional analysis and logistic regression models for permanent pacemaker implantation by glomerular filtration rate

	Glomerular filtration rate < 30 ml/min (*n* = 44)	Glomerular filtration rate 30–60 ml/min (*n* = 58)	Glomerular filtration rate > 60 ml/min (*n* = 75)
Permanent pacemaker implantation			
Number of patients	6	12	31
Case rate, %	13.6	20.7	41.3
Permanent pacemaker implantation			
HR (95% CI)			
Model 1: unadjusted	1 [Referans]	1.65 (0.62–4.40)	3.64 (1.51–8.74)
Model 2: adjusted for medications	1 [Referans]	1.53 (0.51–6.52)	3.32 (1.35–9.38)
Model 3: adjusted for all covariates^a^	1 [Referans]	1.35 (0.49–6.99)	3.00 (1.42–12.10)

*CI* confidence interval, *HR* hazard ratio

^a^Adjusted for; > 1 drug usage, medications and first measurement during hospitalization of the following laboratory values (creatinine, urea, peak troponin, potassium)

## Discussion

In the study, 177 patients who were hospitalized with drug-related AV block and did not require PPI during hospitalization were evaluated. In the cases, beta-blockers were the most frequent responsible drug group and non-dihydropyridine group calcium channel blockers were the second frequent responsible drug group for AV block. In 27.6% of the cases resulted in permanent PM implantation in the long-term follow-up although responsible drug was not continued in the follow-up after discharge. A GFR above 60 ml/min is an independent significant risk factor in predicting long-term permanent pacemaker implantation in drug-related AV blocks. The need for permanent PM implantation in follow-up after discharge is 1.9 times higher in patients with a GFR above 60 ml/min during hospitalization compared to those with a GFR below 30 ml/min during hospitalization.

Drugs affecting the AV conduction system such as beta blockers, non-dihydropyridine group calcium channel blockers (verapamil, diltiazem), amiodarone and digoxin are common etiologies of acquired AV block in clinical practice. Although it is common in clinical practice, our knowledge about the clinical course and prognosis of drug-related AV block is limited.

In a study by Zeltser et al. 169 AV block cases were evaluated. 54% of the cases used beta blocker and/or verapamil or diltiazem. Improvement in AV conduction was observed in 41% of the cases after the cessation of the responsible drug. In 56% of the cases with improvement in AV conduction, it was observed that AV block recurred within 3 weeks, although responsible drug was not given [5].

In a study by Osmonov et al. 668 cases of second-degree AV block, third degree AV block, 2: 1 AV block, atrial fibrillation with bradyarrhythmia were evaluated. Beta blockers and digoxin are the most frequent drug groups that are responsible for AV Blocks. In 72% of the cases, AV block was resolved after the responsible drug was discontinued. A resolution was observed in 6.6% of non-drug-related AV block. Recurrence was observed in 27% of the AV block patients who had improvement in the AV conduction system, although the responsible drug was not continued [6]. In our study, although responsible drug was discontinued in 27% of the cases, AV block recurred after an average of 10 months and the patients needed permanent pacemaker implantation.

In a study by Bozorgi et al. 316 drug-related AV block and 807 cases of AV block due to other etiological reasons were evaluated. While AV conduction improved in 12% of drug-related AV block patients after responsible drug withdrawal, this rate was 6% in AV block cases due to other etiological reasons. Recurrence was observed in 18% of drug-related AV block cases and 40% in AV block cases due to other etiological reasons [7].

923 cases of type 2 diabetes mellitus were evaluated in a study by Mantovani et al., it was seen that the decrease in eGFR was an independent risk factor for the development of cardiac conduction system disorders in patients with type 2 diabetes mellitus. The pathophysiological mechanism of this relationship has not been clearly demonstrated. Endothelial dysfunction, coagulation-fibrinolytic system abnormalities, low grade inflammation, increased oxidative stress and hyperuricemia may affect myocardium and His-Purkinje conduction system structurally and electrophysiologically and making the patient susceptible to cardiac conduction disorders [2].

It can be considered clinically that drug-related AV block has a good prognosis and discontinuation of the responsible drug will improve AV conduction system; however as shown in studies and this study, it was observed that AV block recurred in follow-up of some cases, despite the discontinuation of the responsible drug. Underlying AV conduction system disorders in cases may be manifested clinically with drugs affecting the conduction system. Although improvement appears in the AV conduction system after drug discontinuation, the underlying conduction system disorder may become evident again after a certain period of time. The true etiology of AV block in drug-related AV block has not been fully elucidated yet.

As demonstrated in our study alone, chronic renal failure is not an independent predictor of drug-induced AV block, but high GFR is a predictor which was considered to be secondary to degeneration of the conduction system. Cardiac fibrosis causes stiffening of the cardiac tissue and affects the cardiac conduction system. At the cellular level, activation of the cardiac stromal cells and myofibroblast formation and at the molecular level, transforming growth factor β (TGF-β) are considered as main regulators of the profibrotic processes. Loss-of-function mutations in SCN5A cause myocardial interstitial fibrosis via TGF-ß pathway. With these and other yet unexplained mechanisms, degeneration in the cardiac conduction system may start at an early age and drug use in these patients may manifest the degeneration in the conduction system. It may play a role in the development of drug-induced AV block in patients with high GFR. Although the responsible drug was discontinued in our study population, AV block has recurred in the following months. The potential underlying mechanism responsible for PPI need was considered to be progressive degeneration. Prospective studies evaluating the conduction disturbances with EPS are warranted to enlighten the degeneration of the conduction system [8, 9].

Our knowledge about the effect of electrolytes, especially potassium, on drug-induced AV blocks is limited. The intracellular level of electrolytes and at what levels the cardiac conduction system is affected are not clearly known. Potassium excretion is impaired in patients with chronic kidney failure and blood potassium level may increase. Excessive potassium consumption in these patients or the development of metabolic acidosis due to chronic renal failure contributes to the increase in blood plasma potassium level. Development of hyperkalemia may also lead to blockage in the AV conduction system in patients. Electrolyte disturbances that may develop intracellular and/or extracellular in patients with chronic renal failure may predispose to AV block. It may be thought that patients with normal GFR have less intracellular electrolyte imbalance [4, 10, 11].

In patients with chronic renal failure, the plasma half-life time of drugs excreted by the kidneys may vary. Bisoprolol, one of the beta blocker molecules, commonly used in cardiology daily practice, is excreted from the body 50% through the liver and 50% through the kidneys. Atenolol and sotalol are excreted from the kidneys without any change in the molecule. 5% of the metoprolol is excreted unchanged, the remainder is excreted by the kidneys through its metabolites. Organ failure in the liver or kidneys leads to an increase in the plasma half-life of the beta blocker molecule; thus, even at therapeutic doses, the plasma beta blocker molecule level can be higher than normal [3, 12]. It may be thought that patients with normal GFR have less increased level of drug molecules.

The development of drug-related AV block in patients with normal GFR may be an indicator of an impairment in cardiac conduction pathways compared to ones with low GFR in the long-term follow-up since high GFR was found to be an independent predictor of PPI in patients with drug-related AV block.

### Study limitations

The study was designed as a single center and patients were evaluated retrospectively. Except for the drug groups evaluated in the study, the drug groups in the AV block etiology are not evaluated, so the results of the study cannot be generalized for all drug groups. EPS has not been performed to evaluate cardiac conduction systems during admission or follow-up.

## Conclusions

Recurrence of AV block is high in the follow-up of patients whose AV conduction system has improved by discontinuing the responsible drug in drug-related AV blocks. The development of drug-related block in patients with normal GFR can be considered as an indicator of the disease in the cardiac conduction pathways.

## Data Availability

The datasets used and/or analyzed during the current study are available from the corresponding author on reasonable request.

## Abbreviations

AV: Atrioventricular
EPS: Electrophysiological study
GFR: Glomerular filtration rate
PM: Permanent pacemaker
PPI: Permanent pacemaker implantation
